# Acute Cough Management in Persian Medicine

**DOI:** 10.31661/gmj.v9i0.1854

**Published:** 2020-12-26

**Authors:** Ali Jabbari Sabbagh, Farshad Mohammadian Rasanan, Ahmad Bahrami, Bagher Minaie Zangii, Jale Aliasl, Mekyal Rambod, Omid Sadeghpour

**Affiliations:** ^1^Research Institute for Islamic and Complementary Medicine, Iran University of Medical Sciences, Tehran, Iran; ^2^School of Persian Medicine, Iran University of Medical Sciences, Tehran, Iran; ^3^Student Research Committee, Iran University of Medical Sciences, Tehran, Iran; ^4^Division of Allergy and Immunology, Ali-Asghar Hospital, Iran University of Medical Sciences, Tehran, Iran; ^5^Department of Histology, School of Medicine, Tehran University of Medical Sciences, Tehran, Iran; ^6^Traditional Medicine Clinical Trial Research Center, Shahed University, Tehran, Iran; ^7^Pediatrician, Private Clinic, Tehran, Iran

**Keywords:** Cough, Herbal Remedies, Traditional Medicine

## Dear Editor,

Cough is one of the most common causes of referral to medical centers [1]. The acute cough usually lasts less than three weeks and can be caused by a viral infection and common cold [2]. Cough is a protective mechanism for the airway and helps clear the airway of excess secretions and foreign particles [1]. Although acute cough is usually transient and self-limiting, it can lead to sleep disturbances, experience musculoskeletal pain, and urinary incontinence [3], so treatment of acute cough is essential. There are three categories of drugs for cough management includes expectorants, cough suppressants, and antihistamines [4]. According to studies, there is no good evidence for the effectiveness of these drugs against acute cough [5]. Also, codeine-based medications are currently not recommended for children under 12 with respiratory conditions [6]. Therefore, the use of other effective alternatives therapy, such as herbal medication, is necessary.

Nowadays, herbal medicines are popular because people believe these drugs have a natural origin and fewer side effects [7]. Persian medicine (PM) has defined a specific classification and cough treatment [8-10]. In PM, causes of cough include cold weather, cold water, warm weather, dry weather, smoke, and dust, the taste of sour or spicy or astringent foods and like aspiration while speaking and /or causes that create connection disorders in the lungs like drugs making a wound in the lungs [11]. PM presents the nutrition plan and herbal remedies for the treatment of acute cough. In PM, the nutrient has an essential role in preventing and treating all of the disease [8]. A nutritional plan for dry cough treatment is included barley soup, soft-boiled eggs, bean flour porridge and sugar, fresh butter with sugar, Halva with almond oil, Low-fat rice with chickens, Mung bean rice. Allmond milk, currant, and pistachio kernel and its oil, and cow’s milk and oil, the brain cottonseed and dates, honey with half water, Walnuts and carrots, Starch and sugar, Peeled fava bean, Lentils with almonds, Grease the chest and throat with appropriate oil and wax, barley soup. Barley juice and chicken and Pumpkin and sweet pomegranates, Blackberry paste. On the other hand, some food is not useful and should be avoided, such as meat, onion ( *Allium cepa*), date ( *Phoenix dactylifera*), and sour food. Also, herbal remedies have an essential role in acute cough treatment.

In this study, the herbal remedy for acute cough was shown in Table-1. These plants may be used orally, Inhaler, or topically [8-10]. Most of this plant has cold temperament.

Medicinal plants, which are effective on acute cough, are wet and have mucilage. Most herbs introduced to treat cough are safe [12]. The anti-cough effects of some of these herbs have also been confirmed in modern studies [13]. For example, in some studies on *Viola odorata*, *Althaea Officinalis*, and *Rosa damascene*, the antitussive effects of these plants have been investigated and confirmed [13,14]. Codeine that is used to treat a cough is obtained from the *Papaver somniferum* [15]. However, no studies were found on the anti-cough effects of most of these herbs. The interesting and specific point about cough treatment in PM is topical medications, which are especially useful in children. Topical treatment for cough, such as viola oil, is safe and straightforward, especially in children. Clinical studies were suggested to assess the above dietary plan and herbal remedies in patients with acute cough.

## Conflict of Interest

 The authors declare that there is no conflict of interest.

**Table 1:** Herbal Remedy for Acute Cough in Persian Medicine

**Common name **	**Scientific name **	**Persian medicine Name **	**Temperament **	**Method of application**
Viola	*Viola odorata*	*Banafsheh*	Cold and wet	Topically viola oil
Pumpkin	*Cucurbita maxima*	*Gare *	Cold and wet	Orally
Coriander	*Coriandrum sativum*	*Kozboreh*	Cold and dry	Orally
Lettuce	*Lactuca sativa*	*Khas *	Cold and wet	Lettuce water orally
Rosa	*Rosa damascene*	*Ward *	Cold	Rose water inhalation
Quince	*Cydonia oblonga*	*Safarjal*	Cold and wet	Quince Seeds mucilage orally
Flax seeds	*Linum usitatissimum*	*Katan*	Warm and dry	Flax seeds mucilage With candy orally
Jujube	*Ziziphus jujube*	*Onnab*	Cold and wet	Orally
Mallow	Malva sylvestris	*Khobazi*	Cold and wet	Orally
Marshmallow	*Althaea officinalis*	*Khatmi*	Cold and wet	Orally
Psylliom	*Plantago ovata Forsk*	*Bazr -e- ghatona*	Cold and wet	Orally
Poppy	*Papaver somniferum*	*Khashkhash*	Cold and dry	Orally poppy syrup
